# Establishing a physiological environment for visualized *in vitro* brain slice recordings by increasing oxygen supply and modifying aCSF content

**DOI:** 10.1016/j.jneumeth.2009.06.005

**Published:** 2009-10-15

**Authors:** Norbert Hájos, Istvan Mody

**Affiliations:** a Department of Cellular and Network Neurobiology, Institute of Experimental Medicine, Hungarian Academy of Sciences, Szigony u. 43, 1083 Budapest, Hungary; b Department of Neurology, David Geffen School of Medicine, University of California, Los Angeles, CA 90095, USA

**Keywords:** Oscillation, Sharp wave–ripple activity, GABA, Interneuron, Hippocampus, Neurotransmitter

## Abstract

Our insights into the basic characteristics of neuronal function were significantly advanced by combining the *in vitro* slice technique with the visualization of neurons and their processes. The visualization through water immersion objectives requires keeping slices submerged in recording chambers where delivering artificial cerebro-spinal fluid (aCSF) at flow rates of 2–3 ml/min results in a limited oxygen supply [Hájos N, Ellender TJ, Zemankovics R, Mann EO, Exley R, Cragg SJ, et al. Maintaining network activity in submerged hippocampal slices: importance of oxygen supply. Eur J Neurosci 2009;29:319–27]. Here we review two methods aimed at providing sufficient oxygen levels to neurons in submerged slices to enable high energy consuming processes such as elevated firing rates or network oscillations. The use of these methods may also influence the outcome of other electrophysiological experiments in submerged slices including the study of intercellular signaling pathways. In addition, we also emphasize the importance of various aCSF constituents used in *in vitro* experiments.

Ideally, the different aspects of neuronal function should be investigated in the intact brain. However, this aim is difficult to achieve owing to several technical limitations. To overcome some of these problems, acute tissue slices prepared from live brain were introduced to investigate the intra- and extracellular neuronal signaling (Andersen et al., 1977; Schwartzkroin and Andersen, 1975; Skrede and Westgaard, 1971; Yamamoto and McIlwain, 1966). These *in vitro* studies significantly advanced our understanding of the basic principles of information processing in the central nervous system (CNS). Naturally, the maintenance of living cells in tissue slices and keeping them in conditions resembling those found in the intact brain is of paramount importance.

The first chambers developed to study the cellular basis of brain function using tissue slices were of the interface type (Skrede and Westgaard, 1971; Yamamoto and McIlwain, 1966). In interface type chambers (more frequently called the “Oslo” or “Haas” type brain slice chambers) (Haas et al., 1979; Dingledine, 1984; Reid et al., 1988; Steriade, 2001), slices are held on a nylon mesh at the interface between artificial cerebro-spinal fluid (aCSF) and humidified gas (the mixture of 95% O_2_/5% CO_2_), providing adequate conditions for the maintenance of functional living cells and their microcircuits in several hundred-μm-thick brain slices for many hours. In such chambers the nutrient supply from the oxygenated aCSF reaches the slices from the bottom, while a significant portion of the 95% O_2_/5% CO_2_ mixture also diffuses though a thin (50–200 μm) layer of aCSF that covers the slices. The flow rate of aCSF is usually kept low, around 1 ml/min, which means that the full effects of hydrophobic drugs will require at least 30 min of perfusion, to allow for the drug to reach the slice and for its slow diffusion into the tissue (e.g. Thomson et al., 2000). This produces a substantial challenge for the experimenter if a stable control period, a drug effect followed by a washout need to be obtained. But the major disadvantage of the interface type slice chamber is the lack of possibility for high-resolution visualization of the cells and their fine processes.

The technical innovation that combined the electrophysiological recordings and the visualization of cells in slices came in 1989 in thin slices with the use of water immersion objectives (Edwards et al., 1989; Sakmann et al., 1989; Stuart et al., 1993). To visualize the neurons and their fine processes, brain slices are typically placed on a thin transparent plate made of glass or plastic, and are superfused with aCSF, i.e., slices are submerged in the extracellular solution. In submerged slice chambers, brain slices are supplied with gas and nutrients solely through the aCSF using typical flow rates of 2–3 ml/min. This relatively higher flow rate and the submerged nature of the slices allows for the faster exchange of pharmacological agents. Although submerged slice chambers vary a great deal in their shape and the material used for their construction, in every type of submerged chamber slices are superfused only at one of their surface while resting on the other. Under these conditions, concentration gradients for oxygen, nutrients and various chemicals contained in the aCSF develop by default in the slices, which can dramatically affect the experimental results. Not surprisingly, some results obtained in slices maintained in interface type chambers better resembled findings observed in the intact brain, and could not be reproduced in experiments using submerged brain slices. Most differences were observed in experiments where maintaining high levels of neuronal activity was essential (e.g. during network oscillations) (McMahon et al., 1998; Gloveli et al., 2005; Hájos et al., 2009) and in studies of neuronal oxygen deprivations (Croning and Haddad, 1998). These initial observations implied that the oxygen supplies to tissues maintained in interface and submerged slice chambers were considerably different.

## 1 Should the oxygen supply of submerged brain slices be altered?

In the intact brain the vascular system delivers oxygen in a highly controlled manner wherever and whenever is necessary (Vanzetta and Grinvald, 1999; Vanzetta et al., 2005). In contrast, in brain slices where the vascular system is not functional, the oxygen supply of neurons is limited by the diffusion from the tissue environment (Pomper et al., 2001). Thus, *in vivo* the oxygen supply is modified on demand depending on the local neuronal activity, whereas *in vitro* the experimenter sets a constant oxygen concentration that is difficult to change. Although the results of some electrophysiological investigations obtained in slices are not significantly affected by the amount of oxygen supplied (e.g. evoked potentials; Huchzermeyer et al., 2008), other neuronal functions critically depend on high energy consumption, and accordingly on the amount of oxygen supply. For instance, gamma (30–100 Hz) oscillations, synchronous network activities that emerge from the rhythmic discharges of large neuronal ensembles (Csicsvari et al., 2003; Mann et al., 2005), consume a significant amount of energy (Huchzermeyer et al., 2008). Such oscillations, however, could only be recorded transiently in submerged slices using flow rates of 2–3 ml/min (McMahon et al., 1998; Gloveli et al., 2005; Hájos et al., 2009). These findings imply that the oxygen supply to slices maintained in submerged recording conditions is inferior compared to the conditions of interface chambers and those of the intact brain (Reid et al., 1988). Differences between slices and the intact brain in oxygen availability during neuronal function have been discussed in detail in a recent review (Turner et al., 2007).

In this paper we show that not only network oscillations depend on oxygen concentration supplied to the submerged slices, but other critical experiments might also be affected by the recoding conditions. First, we will present some technical solutions to help increase the oxygen supply of submerged slices.

## 2 Improving the oxygen supply of submerged brain slices

There are at least two methods to improve the oxygen supply of slices maintained in a submerged chamber. First, if slices are being superfused only at one surface, the volume of the submerged chamber should be reduced as much as possible and the flow rate of superfused aCSF should be considerably increased. Second, if the slices can be placed on a mesh with some distance from the supporting plate, the aCSF may be superfused individually at both surfaces of the submerged slices.

We have found that by increasing the flow rate of the aCSF to 3–6 ml/min and reducing the volume of the chamber to 0.5 ml, network oscillations could be readily maintained in hippocampal slices (Hájos et al., 2004; Mann et al., 2005; Oren et al., 2006). In preliminary experiments we found that persistent oscillations in conventional slice chambers designed for visualized patch clamp recordings with volumes of 1–2 ml could only be achieved by increasing the flow rate to >10 ml/min. This is reminiscent of previous observations that persistent network activities in the hippocampal CA3 region (Wu et al., 2005) and spinal cord preparations (Wilson et al., 2003) could only be maintained when using flow rates of 15 ml/min and 22 ml/min, respectively. Thus, the flow rate of the aCSF is a key element in determining the oxygen concentration delivered to the slices. Consequently, higher flow rates can sustain the higher oxygen demand required for synchronous discharges of extensive neuronal ensembles leading to larger oscillatory activities in field potentials. Our recent measurements fully support this assumption (Hájos et al., 2009). Clearly, more oxygen can be delivered by increasing the flow rate, but increasing the rate of slice perfusion has its own technical limitations (e.g. the shape and the volume of the slice chamber, the length and the material of tubing used for perfusion, etc.) (Hájos et al., 2009). The higher perfusion speed may reduce the available time for the diffusion of oxygen through the increased surface of the liquid introduced by the water immersion objective.

**Fig. 1:**
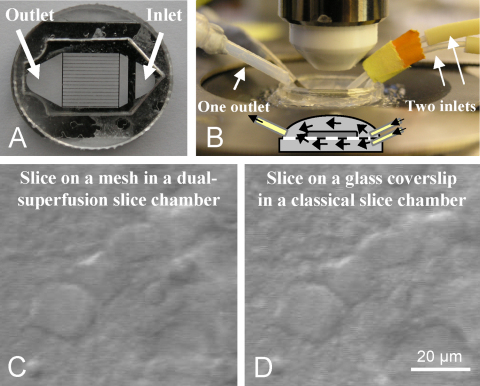
Dual-superfusion slice chamber. (A), Picture of a chamber insert developed for dual-superfusion. The slices are placed on a mesh glued between two plastic rings of a thickness of 2 mm. Solution is separately perfused below and above the slice through two inlets (B). The flow rates and temperatures of the two solutions should be equal to ensure similar conditions at both slice surfaces. Inset in B represents the schematic drawing of the fluid stream in the dual-superfusion slice chamber. Images taken with a CCD camera of the same hippocampal neurons in slices placed on a mesh in a dual-superfusion slice chamber (C), or, for comparison, on a glass coverslip in a classical submerged slice chamber (D). The visibility of neurons and their processes is not compromised in the dual-superfusion slice chamber.

A major drawback of a high flow rate however, is the resulting mechanical instability of the slices, particularly when slice stability is critical for lasting electrophysiological recordings and optical imaging of fine processes. To overcome, or at least to considerably reduce, the problem of slice instability at high flow rates, a dual-superfusion slice chamber may be used, where the slices are placed on a mesh and both surfaces of the slices are individually superfused with aCSF. In this type of slice chamber the mechanical stability of the slices is greatly improved, and the one-sided chemical gradients are significantly reduced, which improves the recording conditions even at relatively low flow rates of 2–3 ml/min (Fig. 1). For more technical details see Hájos et al. (2009).

## 3 Network activity in submerged hippocampal slices

**Fig. 2:**
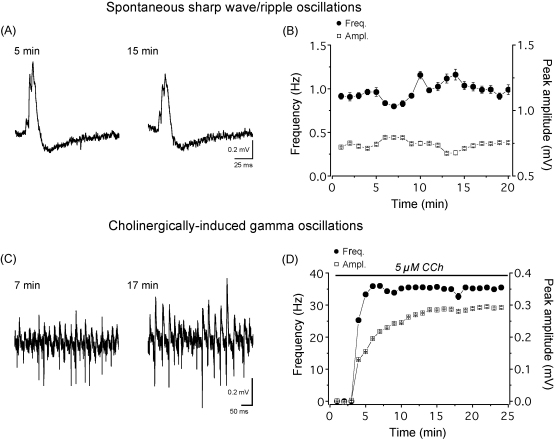
Network oscillations in the CA3 region of mouse hippocampal slices maintained in a dual-superfusion chamber. (A), Sharp wave/ripple oscillations (sample traces taken from the indicated time points) could be readily detected under these recording conditions. (B), the stability of sharp wave/ripple oscillations is shown, where the frequency of their occurrence and their peak amplitudes are plotted as a function of time. (C), Cholinergically induced gamma oscillations could be easily induced in this type of slice chamber using carbachol (CCh) at concentrations as low as 1–5 μM. The raw traces of oscillations were taken from the indicated time points from the plot in (C). (D), Development and stabilization of cholinergically induced gamma oscillations during the wash-in of 5 μM CCh (indicated by horizontal bar) in a plot of the frequency and the peak amplitudes of oscillations as a function of time. In both cases, the network oscillations were recorded in aCSF containing 2 mM Ca^2+^ and 2 mM Mg^2+^ at 32–34 °C. The flow rate was 2–3 ml/min for each channel. Oscillations were recorded with a patch pipette filled with aCSF, placed in the stratum pyramidale. Data are mean ± SEM.

In the intact brain network oscillations, considered to be typical features of neuronal processing, are rhythmic activities generated by the precisely timed discharge of large neuronal populations (Buzsáki, 2006). Oscillations with similar characteristics to those found *in vivo* can be routinely recorded in brain slices maintained in an interface type recording chamber (Whittington et al., 1995; Fisahn et al., 1998; Hájos et al., 2000; Hughes et al., 2004; Lorincz et al., 2008; Maier et al., 2003; Pálhalmi et al., 2004). Yet, network activities in submerged slices are extremely difficult to obtain unless the necessary oxygen supply is provided by elevating the flow rate or by keeping the slices in a dual-superfusion slice chamber. For instance, sharp wave/ripple oscillations known to occur spontaneously in CA3 hippocampal networks *in vivo* (Buzsáki, 2006) and in slices kept in interface type slice chambers (Maier et al., 2003; Buzsáki, 2006) have been readily recorded under these modified submerged conditions (Fig. 2A and B) (Spampanato and Mody, 2007; Hájos et al., 2009). In addition, maintaining pharmacologically induced gamma (30–100 Hz) oscillations in hippocampal slices for extended periods of time (>30 min) also heavily depend on the recording conditions. In submerged chambers with low flow rates, gamma oscillations could be recorded only transiently, whereas at high flow rates these oscillations were maintained just like in recordings in a dual-superfusion slice chamber at lower flow rates (Fig. 2C and D) (Hájos et al., 2004; Hájos et al., 2009). Since pharmacologically induced gamma oscillations could be induced only transiently in submerged slices at low flow rates, yet they were maintained for long periods of time in submerged slices at high flow rates (Hájos et al., 2004; Mann et al., 2005; Oren et al., 2006), in slices kept in interface conditions (Fisahn et al., 1998; Pálhalmi et al., 2004), and in the intact brain (Sakatani et al., 2008), these findings are consistent with the idea that impaired slice oxygen levels might indeed be a limiting factor for network activities in submerged brain slices at low flow rates.

## 4 Single cell synaptic activity in submerged slices

**Fig. 3:**
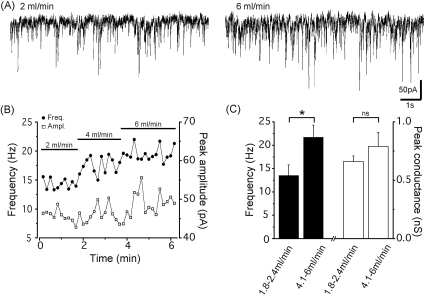
The flow rate determines the spontaneous activity of interneurons as monitored by recording spontaneous inhibitory postsynaptic currents (sIPSCs) in a principal cell. (A), Raw IPSC recordings in a CA3 pyramidal cell using different flow rates. Hippocampal slices prepared from P16–20 rats were maintained in a classical submerged type recording chamber with single superfusion. sIPSCs were recorded by the whole-cell patch-clamp technique in the presence of the ionotropic glutamate receptor blocker kynurenic acid (3 mM) at a holding potential of −65 mV. (B), Plot of the effects of the flow rate on the frequency and the peak amplitudes of IPSCs from the same experiment. (C), At high flow rates, the frequency of sIPSCs recorded in CA3 pyramidal cells was significantly higher (21.7 ± 2.6 Hz, *n* = 9) compared to those recorded at low flow rates (13.5 ± 2.3 Hz, *n* = 11, *p* < 0.05, Student's *t*-test), whereas the average peak conductances of the sIPSCs were similar (low flow rate: 0.66 ± 0.05 nS, *n* = 9; high flow rate: 0.79 ± 0.12 nS, *n* = 11, *p* > 0.1, Student's *t*-test).

In addition to synchronous network events the discharge probability of individual neurons is also affected by oxygen supply in submerged slices. In the absence of any additional pharmacological agents in the aCSF, the frequency of spontaneous inhibitory postsynaptic currents (sIPSCs) recorded in hippocampal slices submerged in a chamber with single superfusion, is significantly higher at high flow rates compared to low flow rates (Fig. 3). There is no difference in the peak conductance of the sIPSCs between the two conditions indicating that more oxygen delivered to slices is vital for the spontaneous firing of hippocampal interneurons in submerged slices. Similarly to the enhanced synaptic inhibition, the oxygen supply can also affect excitatory synaptic transmission. Both the amplitude and the frequency of spontaneous excitatory synaptic potentials (sEPSPs) recorded in CA1 hippocampal interneurons have been found to be significantly larger in slices kept in dual-superfusion chamber compared to those slices, which were placed in a classical chamber with single superfusion at flow rate of 2–3 ml/min (G. Katona, A. Kaszás, G. Turi, B. Rózsa, unpublished observation). The elevated synaptic activity due to the higher oxygen supply might be common in all cortical structures. For instance, in neocortical submerged slices using a flow rate of >8 ml/min, the frequency of spontaneous synaptic currents (both sEPSCs and sIPSCs) has been found to be around 40 Hz recorded in pyramidal cells or in interneurons (Spampanato et al., 2008), values that are substantially higher than those obtained at lower flow rates (∼5–8 Hz, Bandrowski et al., 2003; Yang et al., 2007). These data collectively indicate that the discharge probability of both inhibitory interneurons and pyramidal cells in submerged slices can be varied with oxygen levels. Indeed, it has been observed that more dissolved oxygen in aCSF depolarized the membrane potential and caused a parallel increase in the membrane resistance of CA3 pyramidal neurons (Bingmann et al., 1984), changes that could contribute to the excitability of neurons.

**Fig. 4:**
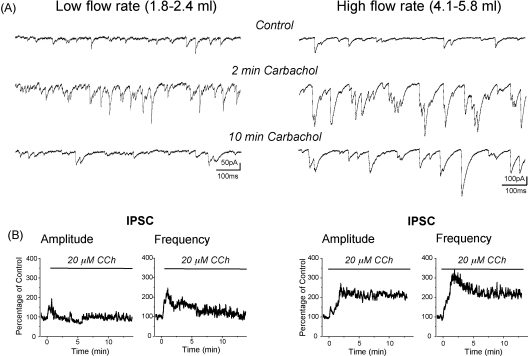
The duration of the cholinergically enhanced inhibitory transmission critically depends on the flow rate in a single superfusion submerged type chamber. (A), Recording of IPSCs before and after carbachol application at low and high flow rates. Measurements were done in rat CA3 pyramidal cells as described in the legend of Fig. 3. (B), Plot of the amplitudes and frequencies of sIPSCs as a function of time at low and high flow rates. The times of the carbachol (CCh) applications are indicated by horizontal bars. Carbachol induced only a transient increase in sIPSC amplitude and frequency at low flow rates. In contrast, increased synaptic inhibition persisted in the presence of carbachol at high flow rates. The values calculated from 5 experiments for both conditions were normalized to control conditions (i.e., before carbachol application).

With the improved spontaneous activity of neurons at rest by elevated oxygen supply, the modulation of neuronal firing by various pharmacological agents might also be altered in submerged chambers when tissue oxygen supply is enhanced. It is well known that in hippocampal slices kept in interface type chambers cholinergic receptor activation (e.g. by carbachol) dramatically increases the spiking activity of inhibitory interneurons. As a consequence, the GABA_A_ receptor-mediated synaptic events recorded in principal cells are enhanced for periods lasting tens of minutes (Pitler and Alger, 1992). A similar lasting increase in synaptic inhibition cannot be observed in submerged slices, unless the recoding conditions are changed. At low flow rates, bath application of carbachol only transiently increases both the amplitude and the frequency of sIPSCs recorded in pyramidal cells of CA3 hippocampal region. In contrast, the carbachol-induced enhancement of synaptic inhibition persists during the whole duration of the perfusion of this cholinergic agonist (Fig. 4) indicating that the high oxygen supply is necessary for the sustained firing of interneurons induced by carbachol.

## 5 Effects of the oxygen supply on intercellular signaling in submerged slices

As shown above, network oscillations, basal and drug-induced firing rates are all affected by the oxygen supply to submerged slices. Therefore, it is reasonable to assume that other critical neuronal events such retrograde signaling at synapses might also be affected by the oxygen levels reaching the slices maintained in submerged conditions. We have recently shown that in the presence of carbachol, nitric oxide and endocannabinoids are critically involved in a form of short-term plasticity at hippocampal GABAergic synapses, the depolarization-induced suppression of inhibition (DSI) (Makara et al., 2007). After comparing the properties of DSI at different flow rates in the presence of carbachol, DSI was more consistently observed in CA1 pyramidal cells at high flow rates (5–6 ml/min; 8/10 DSI) than at low flow rates (2–3 ml/min; 6/19 DSI). Moreover, the magnitude of DSI was also significantly different (at high flow rates: 47.1 ± 13.6%, *n* = 8; at low flow rates: 34.3 ± 6.1%, *n* = 6; *p* < 0.05, Student's *t*-test; N. Hájos, unpublished observations). Since the production of nitric oxide by nitric oxide synthase is affected by the oxygen concentration (Nathan and Xie, 1994), at low oxygen levels caused by low flow rates, the synthesis of nitric oxide might be diminished. Consequently, both the occurrence and the magnitude of DSI would be limited by the oxygen concentration available to the neurons.

To this point, we emphasized the necessity of increasing the oxygen supply of neurons in submerged slices that could help studies of neuronal events under conditions more approaching those *in vivo*. However, the possibility of hyper-oxygenation, which could significantly affect several parameters of neuronal function and may even cause acute cell death (Mulkey et al., 2001; Pomper et al., 2001), should be considered. In the ranges of the flow rates and oxygenation used in our experiments, we have not observed any cell death or neuronal activity that was not also observed *in vivo*. Nevertheless, an optimal range of oxygen supply may need to vary during various recording conditions, and possible unwanted effects of too high oxygen concentrations should also be taken into account.

## 6 Notes on the composition of the aCSF to better approximate physiological conditions

The ionic composition of aCSF used by different laboratories is generally similar with small differences in K^+^, Ca^2+^ and Mg^2+^ concentrations (Reid et al., 1988). These ions are typically added at higher concentrations to the aCSF than they are found in the regular CSF (Di Terlizzi and Platt, 2006). There is also a notable difference between aCSF and CSF in their glucose concentrations. In the CSF, glucose reaches concentration between 1.5 and 5 mM (McNay and Sherwin, 2004), whereas its concentration is kept at 10–25 mM in the aCSF. A difference in glucose availability was shown to affect distinct neuronal functions in slices including network events (Cunningham et al., 2006). Thus, when comparing results from different laboratories it is best to keep in mind that even subtle differences in some of the basic components of the aCSF might impact the outcome of the experiments (Reid et al., 1988).

What about other key ingredients of the natural CSF which are routinely excluded from the aCSF? For instance, neurotransmitter molecules in concentrations sufficient to act through various ionotropic and/or metabotropic receptors are consistently found in the normal CSF (Nyitrai et al., 2006). Neurotransmitter concentrations found in the normal CSF are sufficient to activate tonic conductances in distinct types of neurons by activating high affinity extrasynaptic receptors (Glykys and Mody, 2007). Such molecules are not customarily added to the aCSF, although the activated conductances significantly affect neuronal excitability and network oscillations in slice preparations (Glykys et al., 2008). Since the GABA_A_ receptor-mediated tonic conductance was shown to depend even on the storage conditions of the slices (Glykys and Mody, 2006), it can be assumed that the amount of GABA in slices will considerably vary depending on the slice preparation and maintenance procedures used in various laboratories. Such discrepancies might be ameliorated by adding GABA to the aCSF to yield a final free GABA concentration of 200–500 nM, similar to that found in the normal CSF (Nyitrai et al., 2006). In addition to GABA, glutamate, acetylcholine, and many other known neuroactive molecules are also present in the normal CSF, some of them such as glutamine in mM concentrations (Lerma et al., 1986). Clearly, the concentrations of neuroactive compounds are not steady in the CSF, but are continuously changing as a function of brain activity. For instance, there are dramatic differences in the concentrations of acetylcholine and serotonin during slow wave sleep compared to that found in the CSF of awake animals (Westerink, 1995). Therefore, an argument could be made to use a variety of aCSF with different concentrations of neuroactive compounds to study the equivalent conditions of various brain states *in vitro*.

Neuromodulators like taurine, d-serine, ascorbate, etc. contained in the normal CSF could also significantly impact neuronal signaling if included in the aCSF. Of these compounds, ascorbic acid, an effective controller of free radical levels in the brain and a modulator of cellular excitability and synaptic communication (Rebec and Pierce, 1994), is used more and more often as an additive (1–3 mM) to the aCSF particularly during slice cutting procedure, but rarely in the aCSF used for recordings. In the rat brain CSF the concentration of ascorbic acid is around 0.5 mM, but neurons and glia can accumulate it by Na^+^-dependent transporters (SVCT1 and SVCT2) up to 10 mM and 1 mM, respectively (Rice, 2000). In brain slices the concentration of ascorbate drops to 20% of control levels even after a brief incubation time with ascorbate-free media (Rice, 2000), a reduction that might be prevented by adding ∼0.3 mM ascorbic acid to aCSF. It is reasonable to assume that ascorbic acid is but one of the compounds washed out from slices maintained *in vitro* (see e.g. Kapetanovic et al., 1993), that could significantly affect the experimental results (Table 1.). Therefore, we strongly feel that in order for slice preparations to better resemble the physiological environment of neurons in the intact brain, several compounds should be included in the aCSF. This approach together with ensuring a better oxygen supply to the slices should help the study of the behavior of neurons and their networks as it takes place in the intact brain.

## 7 Concluding remarks

In this review we emphasize the necessity of adequate oxygen supply to submerged slices that might impact the outcome of diverse electrophysiological experiments. Two methods are provided for improving the oxygen supply to submerged slices without significant disturbance of the visualization and recording. We also draw attention to the discrepancy between the components of normal CSF and those of the aCSF used for preparing and maintaining brain slices. We propose including some of the normal CSF constituents in the aCSF and appeal to form a consensus among interested neuroscientists.

## Figures and Tables

**Table 1:** Summary of *in vitro* physiological effects of some CSF components that are not routinely included in aCSF.

CSF components	In CSF (μM)	Preparation	Effects	References
GABA	1–5	Hippocampal slices	Maintaining tonic currents	Glykys and Mody (2006)

Glutamine	400–800	Hypothalamic slice	Increased spontaneous firing	Nishimura et al. (1995)
		Hippocampal slices	Necessary for synaptic function in >4 h, but not in <4 h slices	Kam and Nicoll (2007), An et al. (2008)

Ascorbic acid	500	Forebrain slices	Volume regulation	Brahma et al. (2000)
		Hippocampal slices	Free radical scavenger	Monje et al. (2000)

Taurine	1–10	Hippocampal slices	Volume regulation	Kreisman and Olson (2003)
			Maintained K+ content	
			Higher ATP concentrations in slices	
Lactate	800–2000	Hippocampal slices	Energy supply	Fowler (1993), Schurr et al. (1997); but see Yamane et al. (2000)
Serotonin	1–2	Hippocampal slices	Endogenous release of serotonin from fibers by a 5-HT releaser fenfluramine	Wojtowicz et al. (2009)
